# Estrogen/HER2 receptor crosstalk in breast cancer: combination therapies to improve outcomes for patients with hormone receptor-positive/HER2-positive breast cancer

**DOI:** 10.1038/s41523-023-00533-2

**Published:** 2023-05-31

**Authors:** Mark Pegram, Christian Jackisch, Stephen R. D. Johnston

**Affiliations:** 1 Stanford Cancer Institute, Stanford, CA USA; 2 Obstetrics and Gynaecology and Breast Cancer Center, Klinikum Offenbach GmbH, Offenbach, Germany; 3 Royal Marsden NHS Trust & Institute of Cancer Research, London, UK

**Keywords:** Breast cancer, Tumour biomarkers

## Abstract

The human epidermal growth factor receptor 2 (HER2) is overexpressed in 13–22% of breast cancers (BC). Approximately 60–70% of HER2+ BC co-express hormone receptors (HRs). HR/HER2 co-expression modulates response to both anti-HER2–directed and endocrine therapy due to “crosstalk” between the estrogen receptor (ER) and HER2 pathways. Combined HER2/ER blockade may be an effective treatment strategy for patients with HR+/HER2+ BC in the appropriate clinical setting(s). In this review, we provide an overview of crosstalk between the ER and HER2 pathways, summarize data from recently published and ongoing clinical trials, and discuss clinical implications for targeted treatment of HR+/HER2+ BC.

## Introduction

In population-based studies, human epidermal growth factor receptor 2 (HER2) is overexpressed in ~13–22% of all patients with breast cancer, frequently as a consequence of amplification of the *ERBB2* gene (i.e., clinical “HER2+”)^1–3^, and confers aggressive clinical behavior (particularly a more rapid rate of cell proliferation and propensity for metastases), resulting in poor clinical prognosis^1–5^. Concomitant overexpression of hormone receptors (HRs) and HER2 is also common, with HR-positivity occurring in ~60–70% of patients with HER2+  breast cancer, albeit typically at lower expression levels than in the case of HER2-negative disease^2,4,6–8^. Thus, overall, HR-positive (HR+), HER2+ tumors account for about 10% of all breast cancers^9^. Co-expression of these receptors alters the clinical behavior of tumors^10,11^ and modulates response to both HER2-directed and endocrine therapies (ETs)^12–17^.

Prior to the advent of HER2-targeting therapeutics, patients with HR+/HER2+ breast cancer had significantly worse prognosis compared with HR+/HER2− breast tumors. Clinical trials in the neoadjuvant setting have shown that patients with HR+ tumors have a significantly lower likelihood of achieving a pathologic complete response (pCR) when treated with HER2-targeted therapy plus chemotherapy than those with HR− tumors^15,16,18–22^, whereas in the advanced/metastatic disease setting, those with HER2+ tumors are significantly less responsive to ET than HER2− tumors^17^. In patients with estrogen receptor–positive (ER+) breast cancer, mortality from anti-estrogen–resistant tumors, in part due to aberrant intracellular signal transduction events as a consequence of constitutive activation of the HER2 kinase in HER2+ disease, accounts for a disproportionate fraction of breast cancer mortality each year, despite initial efficacy with selective ER modulators, selective ER downregulators, or estrogen suppressive therapies^23^. As for breast cancers that are both ER+ and HER2+, inhibition of HER2 alone enables potential compensatory escape pathways due to the bi-directional signaling (i.e., crosstalk) between these receptor signaling pathways, which can result in resistance to HER2-directed agents and eventual tumor growth^8,24–28^. In addition, a significant and inverse association has also been observed between *quantitative* measurements of *ERBB2* gene copy number and ER/progesterone receptor (PR) protein expression levels in breast cancer, which may further explain the relative resistance of HR+/HER2+ tumors to ETs^8^. However, although HR expression is quantitatively lower in HER2+ tumors, that is not to say that steroid HRs are inactive. The constitutive activation of HER2 due to overexpression results in activation of downstream signaling cascades that can lead to post-translational modification (phosphorylation) of the ER, rendering ER constitutively active, indeed even ligand-independent^29^. However, despite substantial crosstalk between HER2 and ER and PR signaling pathways, the presence of HR expression in patients with HER2+ early-stage breast cancer remains a predictive biomarker for response to ET^30,31^, and both ERs and HER2 receptors prevail as drivers of tumor growth.

As ER-HER2 crosstalk has been implicated in the development of resistance to both endocrine and HER2-directed agents^32–34^, published preclinical and clinical research suggest a potentially greater benefit for dual- versus single-receptor targeting of HR+/HER2+ breast cancer^7,23,35,36^. Benefits of such combinations have also suggested potential efficacy in treatment regimens without chemotherapy, which may reduce treatment burden and adverse events (AEs) as well as improve patient quality of life^37,38^. In this article, we provide an overview of preclinical evidence of crosstalk between the ER and HER2 cell signaling pathways, discuss the implications for targeted treatment of HR+/HER2+ breast cancer, and review new clinical evidence from randomized trials demonstrating enhanced antitumor activity with dual ER plus HER2 targeting in breast cancers.

## Dual ER and HER2 signaling: preclinical evidence

**Fig. 1:**
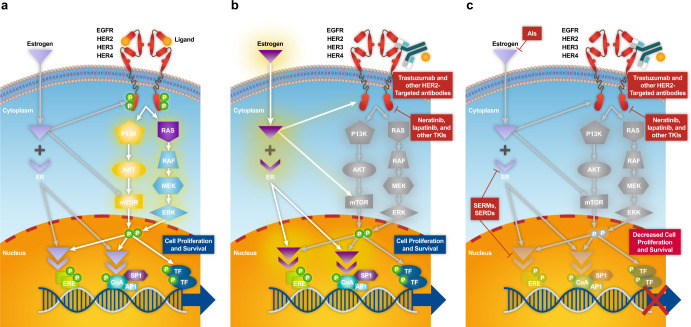
Overview of estrogen receptor (ER) and human epidermal growth factor receptor 2 (HER2) signaling crosstalk. **a** HER2 overactivation results in downregulation of ER-regulated transcription and resistance to endocrine therapy. **b** Blockade of HER2 leads to activation of ER gene transcription via signaling crosstalk pathways as a compensatory mechanism for tumor growth and survival. **c** Inhibition of HER2 and ER pathways are both required to achieve effective HER2+/HR+ antitumor activity. Gray shading of the PI3K/AKT/mTOR and RAS/RAF/MEK/ERK signaling pathways in panels a and b indicate downregulation of these pathways. AI aromatase inhibitor, ER estrogen receptor, ERK extracellular signal-regulated kinase, HER2 human epidermal growth factor receptor 2, HR hormone receptor, MEK mitrogen-activated protein kinase kinase, mTOR mammalian target of rapamycin, P13K phosphatidylinositol 3-kinase, RAF rapidly accelerated fibrosarcoma, SERD selective estrogen receptor degrader, SERM selective estrogen receptor modulator, TKI tyrosine kinase inhibitor.

A wealth of preclinical evidence highlights the role of ER and HER2 crosstalk in the development of resistance to both endocrine and anti-HER2 therapies, thus supporting the rationale for combined receptor blockade targeting the ER and HER2 as a treatment approach in breast cancer^32–34^. ER and HER2 are mediators of two key pathways implicated in the pathogenesis of breast cancer^39^. Bi-directional signaling, or crosstalk, between the ER and epidermal growth factor receptor (EGFR)/HER2 signaling pathways, summarized in Fig. 1, has been implicated in tumor growth and survival as well as the development of resistance to ET in HR+/HER2+ tumors.

The classical function of ER is its nuclear or genomic activity, whereby ER-regulated gene transcription of ER-regulated genes promotes cell proliferation and survival^32,33,40^. In HR+ tumors, aberrant signaling of the ER pathway results in upregulation of ER–regulated gene transcription and subsequent tumor growth. In HR+/HER2+ tumors, bi-directional crosstalk between the ER pathway and HER2 signaling via the downstream RAS and phosphatidylinositol 3-kinase (PI3K) pathways, and/or downregulation of HR expression, can lead to the loss of sensitivity to ET. Hyperactive signaling of the HER2 pathway, resulting from *ERBB2* gene amplification, protein overexpression, or even activating mutations (for example in the *ERBB2* sequence encoding the kinase domain), activates downstream kinases including Akt and mitogen-activated protein kinases (MAPKs)^26^. This, in turn, results in attenuation of the expression of ER messenger RNA (mRNA) and protein and subsequently downregulates ER-regulated gene transcription, resulting in a decrease in sensitivity to ET (Fig. 1a). Preclinical cell line models of ER+ breast cancer have revealed that HER2 overexpression or HER family activation via addition of recombinant heregulin β1—a HER family ligand—resulted in attenuated ER expression, with no impact on intrinsic estradiol-binding affinity to the ER by Scatchard analysis^36^. This mechanism presumably accounts for the observation that in a large cohort of patients with primary breast cancer (*N* = 894), *ERBB2* gene copy number (and resulting HER2 protein overexpression) was inversely correlated with ER and PR protein expression quantified by enzyme immunoassay or a radioligand-binding assay (by Scatchard analysis), thus validating seminal preclinical experimental predictions using a large number of actual human breast cancer clinical specimens^8^.

Therapeutic blockade of HER2 signaling in HER2+ breast cancer can lead to the upregulation/reactivation of ER-regulated gene transcription or increased ER dependency as shown in Fig. 1b. Bi-directional signaling crosstalk can function as a compensatory mechanism for tumor growth and survival, resulting in acquired treatment resistance^40^. In a cell line model of HER2-overexpressing breast cancer, chronic exposure to the tyrosine kinase inhibitor (TKI) lapatinib (a dual-targeted quinazoline small-molecule inhibitor that reversibly binds to the cytoplasmic ATP-binding sites of EGFR/HER1 and HER2 receptors, thereby blocking tyrosine kinase enzymatic activity)^41^ led to development of acquired resistance mediated by increased ER signaling and a switch to co-dependency on ER and HER2^42^. Importantly, in clinical/translational investigations, these authors also observed increased ER signaling in tumor biopsies from lapatinib-treated patients with HER2-overexpressing breast cancer^42^. Reactivation of ER signaling was also evident following the initial administration of lapatinib in some HER2+ breast cancer cell lines, which was followed by reversion to dependence on HER pathways during prolonged exposure to lapatinib^43^. In addition, crosstalk between ER and HER2 has also been shown to upregulate MYC-mediated glutamine metabolism, thus promoting cell proliferation and leading to aromatase inhibitor (AI)-resistant breast cancer cells^44^. The mechanism proposed for this finding is based upon the fact that: 1) the MYC oncogene is an estrogen-dependent gene, transcriptionally regulated by ER in AI-resistant cells^45^; 2) upregulation of MYC was shown to be HER2-dependent (specifically via MAPK signaling and constitutive activation of ER); 3) MYC enhances glutamine uptake from the extracellular space in AI-resistant breast cancer cells through upregulation of the glutamine importer, solute carrier family SLC1A5; and 4) glutaminase, the enzyme responsible for converting glutamine into glutamate, is also regulated by MYC^44^. Taken together, this work demonstrates that MYC is upregulated by the crosstalk between ER and HER2 in AI-resistant breast cancer cells and that MYC-mediated glutamine metabolism is associated with AI resistance of breast cancer^44^.

These findings further support the hypothesis of bi-directional crosstalk between the ER and HER2 pathways in breast cancer, which can lead to the activation of an alternative “escape” survival pathway (e.g., ER signaling) that gives rise to the development of acquired treatment resistance. More extensive blockade of multiple pathways, such as the use of combination therapies for dual blockade of the HER2 and ER pathways in HR+/HER2+ tumors (Fig. 1c), may be necessary to overcome treatment resistance and sustain antitumor activity.

Accordingly, further investigation and research have been conducted to evaluate the benefit of extended dual ER/HER2 blockade and HER2 receptor inhibition. To date, dual blockade of ER/HER2 signaling using combinations of agents that directly target ER (e.g., selective estrogen receptor degraders [SERDs], selective estrogen receptor modulators [SERMs], AIs) and HER2 (e.g., monoclonal antibodies [mAbs] or small-molecule TKIs) have proven to be highly effective in preclinical models (cataloged in Table 1)^23,41,42,46–55^.

**Table 1:** Preclinical data in breast cancer cell lines and xenografts.

Article	ER-targeting agent	HER2-targeting agent	Models used	Key summaries
**In vitro studies**				
Rusnak 2001^41^	None	GW2016 (Lapatinib;TKI)	Activity in *ERBB2*− overexpressing cell lines, including breast cancer cell line (BT474)	Lapatinib inhibited tumor xenograft growth of BT474 cells at doses of 30 and 100 mg/kg, with complete inhibition at the higher dose
Xia 2006^42^	Fulvestrant (SERD)	Lapatinib (TKI)	Acquired resistance to lapatinib in *ERBB2* (HER2)-overexpressing/ER+ breast cancer cell line (BT474)	Acquired resistance to lapatinib was mediated by a switch to ER and HER2 codependence rather than loss of HER2 expression or insensitivity of HER2 signaling to lapatinib Treatment with fulvestrant plus lapatinib effectively prevented the outgrowth of viable resistant cells
Cavaliere 2010^46^	Formestane (anti-aromatase)	Trastuzumab (mAb)	CD44+/CD24-low breast cancer cells from epithelial-mesenchymal co-cultures of human breast cancer tissue	Treatment with trastuzumab, formestane, or formestane + trastuzumab were associated with 16% (*P* < 0.01), 25% (*P* < 0.01), and 50% (*P* < 0.001) inhibition of cell growth, respectively Cell growth inhibition correlated with decreased levels of cyclin D1
Chan 2017^47^	Endoxifen (non-steroidal SERM)	Lapatinib (TKI)	Panel of breast cancer cell models of ERα/HER2 crosstalk that exhibiting either a priori ERα/HER2 crosstalk (BT474) or acquired ERα/HER2 crosstalk (TAM-R, MCF-7/HER2)	Synergistic anticancer effects for lapatinib plus endoxifen were observed in all models of ERα/HER2 crosstalk
Collins 2017^48^	Fulvestrant (SERD)	Pertuzumab (mAb), lumretuzumab (mAb)	Human ER+/HER2-low breast cancer cell line (MCF-7)	Lumretuzumab and pertuzumab were both potent inhibitors of HER2/HER3 signaling in MCF-7 cells when administered as single agents
Li 2015^49^	Fulvestrant (SERD)	Lapatinib (TKI)	Acquired resistance to lapatinib in breast cancer cells (BT474)	In lapatinib-resistant BT474 cells, significant inhibition of PI3K/Akt pathway and activation of MAPK and ER pathways were detected Cell growth was markedly suppressed by the lapatinib plus fulvestrant
McDermott 2017^50^	Tamoxifen (SERM)	Trastuzumab (mAb)	Models of HER2+/ER+/IGF-1R+ breast cancer (MCF7, BT474, MDA-MB-361, HCC1419)	Dual blockade of ER and IGF-1R enhanced growth inhibition in all 4 HER2+ cell lines and increased cell cycle arrest in G1 in BT474 cells Enhanced responses were also observed when trastuzumab + tamoxifen was combined with an IGF-1R TKI Acquired resistance to trastuzumab may be overcome by tamoxifen plus an IGF-1R TKI
Croessmann 2018^23^	Fulvestrant (SERD)	Neratinib (TKI)	ER+ MCF-7 breast cancer cells with isogenically incorporated *ERBB2* kinase domain mutations	Resistance to estrogen deprivation and fulvestrant were observed The combination of fulvestrant and neratinib was associated with potent inhibition of fulvestrant-resistant breast cancer cells that harbor *ERBB2* kinase missense mutations
Sudhan 2018^51^	Fulvestrant (SERD)	Neratinib (TKI)	ER+/HER+ breast cancer cell lines (BT474, MDA-MB-361, and UACC-893)	The combination of neratinib + fulvestrant was associated with inhibition of ER/HER2 crosstalk and ER+/HER2+ breast cancer cell growth
Ribas 2018^53^	Fulvestrant (SERD) Tamoxifen (SERM)	Neratinib (TKI)	Panel of ER+ breast cancer cell lines adapted to long-term estrogen deprivation, expressing wild-type or mutant *ESR1*, and modeling acquired resistance to an AI	Concentration-dependent decreases in proliferation were observed with neratinib or everolimus alone, irrespective of *ESR1* mutation status Combination therapy with neratinib plus endocrine therapy or everolimus plus endocrine therapy further reduced proliferation, but the maximal inhibitory effect was observed with the triple combination of neratinib + everolimus + endocrine therapy
Nayar 2019^54^	Fulvestrant (SERD)	Neratinib (TKI)	ER+/HER2+ breast cancer cell lines (MCF-7 and T47D) transfected with constructs encoding various *ERBB2* mutants or controls	*ERBB2* mutations conferred estrogen independence and resistance to tamoxifen, fulvestrant, and palbociclib Neratinib overcame endocrine resistance in *ERBB2*-mutant, ER+ breast cancer cell lines and provided enhanced tumor growth inhibition when combined with fulvestrant
Shagisultanova 2019^55^	Fulvestrant (SERD)	Tucatinib (TKI)	HR+/HER2+ breast cancer cell lines (BT474, MDA-MB-361, and UACC-812)	The combination of fulvestrant, tucatinib, and palbociclib was active in all 3 cell lines; addition of fulvestrant to tucatinib + palbociclib improved the activity of the doublet Tucatinib and its doublet and triplet combinations suppressed downstream effector phERK1/2 in BT474; only doublet and triplet combinations were effective in MDA-MB-361
**In vivo studies**				
Scaltriti 2016^52^	g (SERD)	Neratinib (TKI)	Cell-based and patient-derived ER+, HER2-driven xenograft models	Neratinib plus fulvestrant significantly inhibited tumor growth and prolonged survival in tumor-bearing mice, compared with single-agent or control treatments
Collins 2017^48^	Fulvestrant (SERD)	Pertuzumab (mAb), lumretuzumab (mAb)	Mouse xenograft model of ER+/HER2-low/HER3+ human breast cancer (HBCx-19)	Treatment with single-agent lumretuzumab and pertuzumab induced tumor stasis (100% RTV) and partial regression (60% RTV) Pertuzumab + lumretuzumab was associated with strong tumor regression (10% RTV) Triple combination therapy with pertuzumab + lumretuzumab + fulvestrant was the most efficacious treatment, inducing long-lasting tumor regression
Li 2015^49^	Fulvestrant (SERD)	Lapatinib (TKI)	BT474 xenograft model of acquired resistance to lapatinib	Combination therapy with lapatinib + fulvestrant was associated with a significantly greater reduction in tumor volume, compared with either agent alone
Croessmann 2018^23^	Fulvestrant (SERD)	Neratinib (TKI)	Xenograft models of *ERBB2* mutant breast cancer (mice injected with MCF-7 cells harboring *ERBB2* mutations)	*ERBB2* mutations hyperactivate the HER3/PI3K/Akt/mTOR axis, leading to anti-estrogen resistance in ER+ breast cancer cells Treatment with neratinib + fulvestrant restored sensitivity to fulvestrant; dual blockade of HER2 and ER pathways was needed for treatment of ER+/*ERBB2* mutant breast cancers
Sudhan 2018^51^	Fulvestrant (SERD)	Neratinib (TKI)	ER+/HER2+ xenograft models of breast cancer (mice injected with MDA-MB-361 cells)	Extended adjuvant therapy with fulvestrant + neratinib maintained complete responses, whereas fulvestrant alone resulted in rapid relapse
Ribas 2018^53^	Fulvestrant (SERD) Tamoxifen (SERM)	Neratinib (TKI)	Xenograft model of resistance to AI therapy (BLAB/c FOX nude mice injected with MCF-2a-long-term estrogen deprivation cells)	Triple combination with neratinib + everolimus + fulvestrant was the most effective treatment for reducing tumor volume

Italic text indicates gene names.

*AI* aromatase inhibitor, *ER* estrogen receptor, *HER2* human epidermal growth factor receptor 2, *HR* hormone receptor, *IGF-1R* insulin-like growth factor 1 receptor, *mAb* monoclonal antibody, *mTOR* mammalian target of rapamycin, *PI3K* phosphatidylinositol 3-kinase, *RTV* relative tumor volume, *SERD* selective estrogen receptor degrader, *SERM* selective estrogen receptor modulator, *TKI* tyrosine kinase inhibitor.

Pietras and colleagues^36^ were the first to demonstrate that combined receptor blockade using an anti-HER2 antibody and tamoxifen resulted in greater combined efficacy compared to either the anti-HER2 antibody or anti-estrogen–alone controls against HER2-overexpressing human breast cancer cells co-expressing ER. Working independently some years later, Xia and colleagues^42^ reported in vitro experiments in HR+/HER2-overexpressing breast cancer cell lines and tumor xenograft models confirming the rationale for dual ER/HER2 blockade, this time with lapatinib plus fulvestrant to enhance efficacy (vs. monotherapy with either agent alone as controls), using a different HER2-targeting agent and different model systems, yet completely consistent with the earlier findings published by Pietras^36^. Combination therapy with a HER2-targeted agent and ET gave rise to more complete tumor regression than HER2-targeted therapy alone in ER+/HER2+ breast cancer cell line and xenograft models^43,56–58^. One recent in vitro study demonstrated that tamoxifen- and fulvestrant-resistant breast cancer cell lines were sensitive to treatment with lapatinib or afatinib (a second-generation anilinoquinazoline that irreversibly binds to the intracellular tyrosine kinase domains of EGFR, HER2, and HER4 receptors), and gradual reactivation of ERα sensitivity was observed with lapatinib therapy^34^. Synergistic effects were also evident in this study following combination treatment with afatinib plus an anti-endocrine agent or lapatinib plus tamoxifen or fulvestrant^34^.

It has been suggested that dual blockade of HER2 receptors or blockade of multiple HER receptors may offer enhanced antitumor efficacy^43,56^. Pertuzumab targets HER2 by blocking ligand-dependent HER2-HER3 heterodimerization and reverses HER3-mediated resistance pathways^59^. It has been shown that in HR+/HER2+ breast cancer cell xenografts, the combination of HER1 (EGFR) and HER2 blockade with gefitinib, trastuzumab, and pertuzumab in combination with estrogen withdrawal was more effective in suppressing tumor growth than any of these agents alone^56^. In a separate study, the combination of trastuzumab, pertuzumab, and the HER3-targeted monoclonal antibody patritumab suppressed tumor progression more effectively than trastuzumab alone in a similar breast cancer xenograft model^60^.

Subsequently, novel compounds that irreversibly bind multiple HER receptors have been developed in recent years for the treatment of cancer. One such compound is the oral, irreversible, pan-HER TKI neratinib, which reduces phosphorylation and activation of downstream signaling pathways by covalently binding to a conserved cysteine residue within the ATP-binding pockets of HER1, HER2, and HER4^61^. Initial in vitro experiments demonstrated antitumor activity in both HER2-overexpressing breast cancer and EGFR-overexpressing epidermal carcinoma cell lines, with neratinib inhibiting downstream signal transduction events and cell cycle regulatory pathway, and reducing cell proliferation^62^.

Recent studies have also evaluated neratinib in combination with other targeted agents to overcome treatment resistance in preclinical models of breast cancer^23,52,54^. Dual blockade of ER and HER2 with neratinib plus fulvestrant was associated with significant inhibition of tumor growth and prolonged survival in HR+/HER2+ breast tumor xenografts unresponsive to ET compared with single-agent neratinib or controls^52^. In ER+ breast cancer cell lines and xenografts resistant to anti-estrogen therapies, treatment with neratinib restored fulvestrant sensitivity in cell lines harboring *ERBB2* kinase domain-activating mutations (L755S and V777L), and dual blockade of ER and HER2 with neratinib plus fulvestrant was required to inhibit the growth of these ER+/*ERBB2*-mutant cell lines^23^. In addition, the neratinib plus everolimus and neratinib plus fulvestrant combinations were equally effective at suppressing tumor progression in mice bearing *ERBB2* mutant xenografts and superior to the single-agents–alone controls^23^, emphasizing the importance of targeting both the ER and HER2 pathways in ER+/*ERBB2*-mutant tumors. Furthermore, treatment with neratinib overcame endocrine resistance in ER+/*ERBB2*-mutant breast cancer cell lines, derived from metastatic biopsies from patients with ER+ metastatic breast cancer who developed endocrine resistance to tamoxifen or fulvestrant, thus suggesting enhanced tumor growth inhibition when combining neratinib with fulvestrant^54,63^.

In a human-in-mouse model of ER+/HER2+ breast cancer, adjuvant paclitaxel plus trastuzumab ± pertuzumab for 4 weeks, followed by “extended adjuvant therapy” with fulvestrant plus neratinib for an additional 4 weeks, was associated with maintenance of a complete response (CR), whereas subjects treated with extended fulvestrant alone relapsed rapidly^51^. The combination of neratinib plus fulvestrant was also shown to potently inhibit cancer cell growth; downregulate ER reporter activity, P-AKT, and P-ERK; and reduce cyclin D1 mRNA and protein levels in ER+/HER2+ breast cancer cell lines^51^. Evaluation of neratinib and everolimus in a panel of ER+ breast cancer in vitro and in vivo models of acquired AI resistance demonstrated that the combination of either agent with ET enhanced reductions in cell proliferation and tumor volume, and the greatest effect was observed with the triple combination of everolimus, neratinib, and ET^53^. Achieving durable clinical outcomes in patients with ER+/HER2+ breast cancer may therefore require extended HER2 blockade to overcome reactivation of the HER2 pathway during ER blockade. Further mechanistic insight into perturbation of mammalian target of rapamycin (mTOR) and HER family kinases comes from analysis of gene expression in xenografts following combination therapy with everolimus, neratinib, and ET reduced expression of the EGFR/ERK gene set, which suggests that the EGF/EGFR feedback loop associated with mTOR inhibition was negated^53^.

In another novel approach, resistance to HER2-targeted therapy may be overcome by the addition of cyclin-dependent kinase 4 and 6 (CDK4/6) inhibitors through suppression of Rb phosphorylation and TSC2 phosphorylation that attenuated mTOR complex 1 (mTORC1) activity^64^. This can reduce feedback inhibition of EGFR-family kinases found upstream, allowing for continued treatment of tumors via the EGFR/HER2 blockade^64^. Consequently, inhibition of both EGFR/HER2 and CDK4/6 blockades further suppresses TSC2 phosphorylation, thereby also suppressing mTORC1/S6K/S6RP activity^64^. Combinations of the HER2-targeted small-molecule inhibitor tucatinib, the CDK4/6 inhibitor palbociclib, and fulvestrant were investigated in three HR+/HER2+ human breast tumor cell lines^55^. The tucatinib plus fulvestrant and tucatinib plus palbociclib doublets were synergistic in all three cell lines; the addition of fulvestrant to tucatinib plus palbociclib further improved efficacy in all three cell lines.

Overall, these preclinical laboratory research findings demonstrate that bi-directional crosstalk between the ER and HER2 pathways in breast cancer may lead to the activation of alternative “escape” survival pathways through ER signaling and, eventually, treatment resistance and tumor growth. Therefore, dual blockade of ER and HER2 signaling pathways in HR+ tumors may result in enhanced and sustained antitumor activity. In addition, it has been hypothesized that blockade of multiple HER family receptors may be more effective than blockade of HER2 kinase alone and use of the pan-HER TKI neratinib as part of combination therapy may re-sensitize ER pathways to ET. Such results warranted further investigation in the safety and efficacy of dual ER/HER2 blockade in the clinical setting.

## Dual ER and HER2 targeting: clinical data

Based on the promising preclinical data described above, several clinical trials have evaluated the effectiveness of combining endocrine and HER2-targeted therapies in the neoadjuvant, extended adjuvant, and advanced/metastatic breast cancer settings (Table 2)^7,35,37,38,41,65–97^.

**Table 2:** Clinical data from patients with breast cancer (organized by neoadjuvant, extended adjuvant, metastatic).

Article	Trial type/indication		ER-targeting agent		HER2-targeting agent	Other agent	Key summaries
**Neoadjuvant**							
Masuda 2018^93^	Open label phase II (Neo-LaTH) Invasive breast cancer, neoadjuvant (*n* = 215)		Leuprorelin 11.25 mg (weeks 1-13) + TAM 20 mg/day or letrozole 2.5 mg/day		Lapatinib (1000 mg/day initially, then 750 mg/day), trastuzumab (4 mg/kg in Week 1 and then 2 mg/kg)	Paclitaxel (80 mg/m^2^)	Japanese patients; HER2+ • Lapatinib + trastuzumab followed by lapatinib + trastuzumab + paclitaxel with or without prolongation of anti-HER2, with or without ET ○ 47.9% achieved CpCR (ER−, 63.0%; ER+, 36.1%; *P* = 0.0034) ○ 42.2% achieved pCR with pN0 (ER−, 57.6%; ER+, 30.3%)
Harbeck 2017^91^	Open label phase II (WGSG ADAPT) Early breast cancer, neoadjuvant (*n* = 375)		TAM or AI		T-DM1 (3.6 mg/kg) and trastuzumab (8 mg/kg loading, then 6 mg/kg)	None	HER2+/HR+ • T-DM1 vs. T-DM1 + ET vs. trastuzumab + ET pCR at 12 weeks, 41.0% vs. 41.5% vs. 15.1% (*P* < 0.001)
Prat Aparicio 2017^92^	Open label phase II (PAMELA) Stage I-IIIA HER2+ breast cancer, neoadjuvant (*n* = 151)		Letrozole or tamoxifen		Lapatinib and trastuzumab	None	HER2+ • Overall pCR at 18 weeks was 30.5% (HR+, 18.2%; HR−, 43.2%) • Rate of pCR was 40.6% in HER2-E and 10.0% in non–HER2-E (*P* < 0.0001) • Within HR+ disease, pCR rates were 31.6% in HER2-E and 5.3% in non–HER2-E (*P* = 0.006) Within HR− disease, pCR rates were 46.0% in HER2-E and 27.3% in non–HER2-E (*P* = 0.331)
Gluz 2020^90^	Open label, phase II (WSG TP-II) HR+/HER2+ EBC, neoadjuvant (*n* = 207)		Standard ET		Trastuzumab and pertuzumab	Paclitaxel 80 mg/m^2^	HR+/HER2+ • ET+ trastuzumab + pertuzumab vs. paclitaxel + trastuzumab + pertuzumab • pCR (at 12 weeks), was 24% vs. 57% (*P* < 0.001)
Gianni 2018^69^	Open label phase II (NA-PHER2) Invasive breast cancer, neoadjuvant (*n* = 36)		Fulvestrant 400 mg (SERD)		Trastuzumab (8 mg/kg loading, then 6 mg/kg) and pertuzumab (800 mg loading then 420 mg; mAb)	Palbociclib 125 mg/day (checkpoint inhibitor)	ER+/HER2+ • Change in mean Ki67 expression ○ At BL, 31.9 ○ At 2 weeks, 4.3 (*P* < 0.0001) ○ At time of surgery, 12.1 (*P* = 0.013) • Change in apoptosis from BL to surgery ○ 1.2−0.4 (*P* = 0.019) • Presurgery objective response in 29/30 (97%) patients
Park 2016^78^	Open label phase II (Neo-ALL-IN) Stage II-III breast cancer, neoadjuvant (*n* = 24)		Letrozole 2.5 mg/day (non-steroidal AI)		Lapatinib 1500 mg/day (TKI)	None	Asian patients; ER+/HER2+ tumors • Clinical overall response rate, 62.5% (1 CR; 0 pCR) • Potential biomarkers: TILs, ER expression, IHC ER Allred score, ^18^F-FES PET-CT
Rimawi 2013^71^	Open label phase II (TBCRC 006) Stage II-III breast cancer, neoadjuvant (*n* = 64)		Letrozole (non-steroidal AI)		Lapatinib 1000 mg/day (TKI) and trastuzumab (4 mg/kg loading, then 2 mg/kg; mAb)	None	Stage II-III, HER2+; ER+ (*n* = 40) • In-breast pCR, 27% (ER+ , 21%; ER−, 36%) • Overall pathologic response rate, 49% • Low-volume residual disease rate, 22% (ER+, 33%; ER−, 4%)
Rimawi 2020^94^	Open label phase II (TBCRC023) Stage II-III breast cancer, neoadjuvant (*n* = 97)		Letrozole 2.5 mg/day (non-steroidal AI)		Lapatinib 1000 mg/day (TKI) and trastuzumab (4 mg/kg loading, then 2 mg/kg)	None	Stage II-III, HER2+ (*n* = 97) • 12 vs. 24 weeks of lapatinib + trastuzumab (+ letrozole if ER+) ○ 12-week pCR, 12% (ER+, 9%; ER−, 20%) ○ 24-week pCR, 28% (ER+, 33%; ER−, 18%)
Guarneri 2014^35^	Phase IIb RCT Stage II-IIIA breast cancer, neoadjuvant/adjuvant (*n* = 92)		Letrozole 2.5 mg/day (non-steroidal AI)		Lapatinib 1500 mg/day (TKI)	None	HR+/HER2− • Letrozole + lapatinib vs. letrozole alone ○ Clinical response rate (CR + PR), 70% vs. 63% ○ Ki-67 and pAkt expression were significantly decreased from BL to surgery in both arms ○ ORR in patients with *PIK3CA* mutation, 93% vs. 63% (*P* = 0.04)
Rimawi 2017^76^	Phase III RCT (NSABP B-52) Breast cancer in the neoadjuvant setting (*n* = 315)		AI		Trastuzumab and pertuzumab	Docetaxel, carboplatin, estrogen deprivation (goserelin and AI)	HR+/HER2+ • TCHP + estrogen deprivation therapy vs. TCHP alone ○ pCR (breast and nodes), 46.1% vs. 40.9% (*P* = 0.36) ○ pCR (breast), 47.4% vs. 44.2% (*P* = 0.57)
**Extended adjuvant**							
Chan 2016^7^ Martin 2017^79^ Chan 2021^89^	Phase III RCT (ExeteNET) Stage I-IIIc operable breast cancer, post-adjuvant (*n* = 2,840)		None		Neratinib 240 mg/day (TKI) after trastuzumab (mAb)–based neoadjuvant and adjuvant therapy	None	HER2+ • Neratinib vs. placebo ○ iDFS events at 2 years, 70 vs. 109 events (hazard ratio, 0.67; *P* = 0.0091) ○ iDFS events at 5-year follow-up, 116 vs. 163 events (hazard ratio, 0.73; *P* = 0.0083) ○ DFS rate at 2 (93.9% vs. 91.6%) and 5 (90.2% vs. 87.7%) years ○ DFS benefit with neratinib was observed in HR+ patients, but not in HR− ○ OS rate at 8 years, 91.3% vs 82.2% (hazard ratio, 0.47)
Tolaney 2021^82^	Phase III RCT (eMonarcHER) High-risk breast cancer (planned *n* = 2450)		Standard ET None		Abemaciclib 150 mg (CDK4/6 inhibitor)	None	• Planned to start
Dieci 2022^88^	Phase III (Short-HER) HER2+/HR+ early breast cancer (*n* = 1254)		AI and/or TAM		Trastuzumab	Anthracycline-taxane chemotherapy	HER2+/HR+ • 9 weeks vs. 1 year of adjuvant trastuzumab + anthracycline-taxane chemotherapy + adjuvant ET • 7-year DFS with AI was 87.3% vs. 81.7% with TAM or TAM-AI (hazard ratio, 1.46; log-rank *P* = 0.017)
**Advanced/metastatic**							
Jhaveri 2022^87^	Open label phase II (SUMMIT) Metastatic breast cancer and prior CDK4/6i (*n* = 55)		Fulvestrant 500 mg		Neratinib (240 mg/day), trastuzumab (8 mg/kg initially, then 6 mg/kg)	None	HR+, HER2− • Neratinib + trastuzumab + fulvestrant (*n* = 45) ○ ORR, 38% ○ Median DOR, 14.4 months ○ Clinical benefit, 47% ○ Median PFS, 8.2 months
Ciruelos 2020^86^	Open label phase II (PATRICIA) Advanced breast cancer (*n* = 71)		Letrozole		Palbociclib (200 mg/day), trastuzumab (8 mg/kg loading, then 6 mg/kg IV or 600 mg SC)	None	HER2+ • Palbociclib + trastuzumab (ER−) ○ 6-month PFS, 33.3% • Palbociclib + trastuzumab (ER+) ○ 6-month PFS, 42.8% • Palbociclib + trastuzumab + letrozole (ER+) ○ 6-month PFS, 46.4%
Hua 2022^85^	Open label phase III (SYSUCC-002) Metastatic breast cancer (*n* = 392)		Investigator’s choice ET (ER modulator or AI)		Trastuzumab (8 mg/kg loading, then 6 mg/kg)	Chemotherapy (investigator’s choice of taxanes, capecitabine, or vinorelbine)	Patients in China, HER2+ • Trastuzumab + ET vs. trastuzumab + chemotherapy ○ Median PFS, 19.2 vs. 14.8 months (*P* < 0.0001)
Swain 2020^84^	Phase III RCT (CLEOPATRA) Metastatic breast cancer (*n* = 808)		None		Trastuzumab (8 mg/kg loading, then 6 mg/kg) and pertuzumab (840 mg loading, then 420 mg)	Docetaxel (75 mg/m^2^ escalating to 100 mg/m^2^ if tolerated)	HER2+ • Trastuzumab + docetaxel + pertuzumab ○ Median OS, 57.1 months ○ 8-year OS rate, 37% • Trastuzumab + docetaxel + placebo ○ Median OS, 40.8 months ○ 8-year OS rate, 23%
Chu 2008^74^	Open label phase I Advanced solid tumors (*n* = 34)	Letrozole 2.5 mg/day (non-steroidal AI)			Lapatinib 1250–1500 mg/day (TKI)	None	HR+ (*n* = 18) • In the breast cancer cohort: ○ PR, *n* = 1 ○ SD, *n* = 2 (lasting 247 and 289 days) • Well tolerated • No pharmacokinetic interaction
Fumoleau 2014^77^	Open label phase I Metastatic breast cancer (*n* = 23)	Tamoxifen 20 mg/day (SERM)			Lapatinib 1500 mg/day (TKI)	None	HR+ irrespective of HER2 status • SD, 8/23 (36.4%) • Median PFS, 2.7 months • Well tolerated
Koeberle 2011^75^	Open label phase I AI- and trastuzumab-resistant breast cancer (*n* = 13)	Letrozole (non-steroidal AI)			Trastuzumab (mAb)	None	ER+/HER2+ breast cancer • Trastuzumab alone (step 1) then trastuzumab + letrozole upon disease progression (step 2) • CBR (primary outcome) ○ Step 1, 46% ○ Step 2, 73% • Median TTP 188 days
Shagisultanova 2019^80^	Phase Ib/II trial Metastatic breast cancer (*n* = 20)	Letrozole 2.5 mg/day (non-steroidal AI)			Tucatinib 300 mg BID (TKI)	Palbociclib 125 mg/day (CDK4/6 inhibitor)	• Safety consistent with previous reports • Longest time on trial is 10 months (no CNS disease at BL) and 6 months (CNS disease at BL) Recommended phase II dose is tucatinib 300 mg bid
Marcom 2007^70^	Open label phase II Advanced breast cancer (n = 31)	Letrozole (non-steroidal AI)			Trastuzumab (mAb)	None	ER+ and/or PR+ and HER2+ • Overall response rate, 26% • CBR, 52% • Median TTP, 5.5 months • Median DOR, 20.6+ months
Ma 2021^97^ Ma 2022^83^	Open label phase II (MutHER) Metastatic breast cancer (*n* = 31)	Fulvestrant			Neratinib 240 mg/day (TKI)	None	Patients with prior fulvestrant treatment • ORR, 25% • SD (≥24 weeks), 15% • Median PFS, 24 weeks • CBR, 38% Fulvestrant-naïve patients • ORR, 30% • SD (≥ 24 weeks), 0 • Median PFS, 20 weeks • CBR, 30% Exploratory ER− cohort • CBR, 25%
Villanueva 2013^72^	Open label phase II Metastatic breast cancer (*n* = 24)	Letrozole 2.5 mg/day (non-steroidal AI)			Lapatinib 1500 mg/day (TKI)	None	AI-resistant, HR+ breast • ORR (at 12 weeks), 4% • SD, 25% • CBR, 21% • Median PFS, 3.4 months
Smyth 2020^96^	Open label phase II (SUMMIT) Metastatic breast cancer (*n* = 81)	Fulvestrant 500 mg every 4 weeks (SERD)			Neratinib 240 mg/day (TKI)	None	Neratinib monotherapy vs. neratinib + fulvestrant (70 patients with ER+/HER2+ tumors) • ORR, 17.4% vs. 14.0% • CBR, 30.4% vs. 46.8% • Median PFS, 3.6 vs. 5.4 months
Rimawi 2018^38^	Phase II RCT (PERTAIN) Metastatic or locally advanced breast cancer (*n* = 129)	Anastrozole 1 mg/day or letrozole 2.5 mg/day (non-steroidal AI)			Trastuzumab (8 mg/kg loading, then 6 mg/kg every 3 weeks) and pertuzumab (840 mg loading, then 420 mg every 3 weeks; mAb)	Induction chemotherapy allowed	HR+/HER2+ • Pertuzumab + trastuzumab + AI vs. trastuzumab + AI ○ Median PFS (stratified by induction chemotherapy), 18.89 vs. 15.80 months (hazard ratio, 0.66; *P* = 0.007) ○ CR, 7.3% vs. 0.9% ○ CBR, 68.8% vs. 67.0% ○ Median DOR for CR/PR, 27.10 vs. 15.11 months
Huober 2012^73^	Phase III open label (eLEcTRA) Metastatic breast cancer (*n* = 57)	Letrozole 2.5 mg/day (non-steroidal AI)			Trastuzumab (4 mg/kg loading, then 2 mg/kg; mAb)	None	HR+/HER2+; HR+/HER2−(*n* = 35) • Letrozole + trastuzumab vs. letrozole alone ○ Median TTP, 14.1 vs. 3.3 months (hazard ratio, 0.71; *P* = 0.03) ○ CBR, 65% vs. 39% (odds ratio, 2.99; *P* = 0.0636) ○ ORR,13% vs. 27%
Johnston 2009^65^ Schwartzberg 2010^66^	Phase III RCT (EGF30008) Metastatic breast cancer (*N* = 1,286)	Letrozole 2.5 mg/day (non-steroidal AI)			Lapatinib 1500 mg/day (TKI)	None	HR+/HER2+ (*n* = 219) • Letrozole + lapatinib vs. letrozole alone ○ Median PFS, 8.2 vs. 3.0 months (hazard ratio, 0.71; *P* = 0.019) ○ CBR, 48% vs. 29% (odds ratio, 0.4; *P* = 0.003) ○ ORR, 28% vs. 15% (odds ratio, 0.4; *P* = 0.021) ○ Median OS, 33.3 vs. 32.3 months
Kaufman 2009^67^	Phase III RCT (TAnDEM) Metastatic breast cancer (*n* = 207)	Anastrozole 1 mg/day (non-steroidal AI)			Trastuzumab (4 mg/kg loading, then 2 mg/kg every week) (mAb)	None	HR+/HER2+ • Anastrozole + trastuzumab vs. anastrozole alone ○ Median PFS, 4.8 vs. 2.4 months (hazard ratio, 0.63; *P* = 0.0016) ○ Median OS, 28.5 vs. 23.9 months (*P* = 0.325) ○ 70% of patients in the anastrozole-alone arm crossed over to combination therapy after progression ○ Median TTP, 4.8 vs. 2.4 months (*P* = 0.0007) ○ CBR, 42.7% vs. 27.9% (*P* = 0.026) ○ Median DOR, 9.5 vs. 10.0 months
Johnston 2021^95^	Phase III RCT (ALTERNATIVE) Metastatic breast cancer (*n* = 355)	Letrozole, anastrozole, or exemestane (non-steroidal or steroidal AI)			Lapatinib and trastuzumab (mAb)	None	HR+/HER2+ • Lapatinib + trastuzumab plus AI vs. trastuzumab + AI ○ Median PFS, 11.0 vs. 5.7 months (hazard ratio, 0.62; *P* = 0.0064) ○ Overall response rate, 31.7% vs. 13.7% ○ CBR, 41% vs. 31% • Lapatinib + AI vs. trastuzumab + AI ○ Median PFS, 8.3 vs. 5.7 months (hazard ratio, 0.71; *P* = 0.0361) ○ Overall response rate, 18.6% vs. 13.7% ○ CBR, 33% vs. 31%
Tolaney 2020^37^	Open label phase II (monarchHER) Metastatic breast cancer (*n* = 237)	Fulvestrant 500 mg (SERD)			Trastuzumab (mAb)	Abemaciclib 150 mg (CDK4/6 inhibitor)	• No chemotherapy • Tolerable safety profile • PFS for abemaciclib, trastuzumab, and fulvestrant vs. standard-of-care chemotherapy and trastuzumab (8.3 vs. 5.7 months, 5.4–7.0; hazard ratio, 0.67 [95% CI, 0.45–1.00]; *P* = 0.051)
Zhang 2021^81^	Phase Ib/II trial Metastatic breast cancer (*n* = 15)	Letrozole 2.5 mg/day (non-steroidal AI)			Pyrotinib 400 mg/day (TKI)	SHR6390 150 mg/day (CDK4/6 inhibitor)	• Ongoing • 10 of 15 patients had achieved confirmed PRs

Italic text indicates gene names.

^*18*^*F-FES PET-CT* ^18^F-fluoroestradiol positron emission tomography combined with computed tomography, *AI* aromatase inhibitor, *BID* twice daily, *BL* baseline, *CBR* clinical benefit rate, *CNS* central nervous system, *CpCR* comprehensive pathological complete response, *CR* complete response, *DFS* disease-free survival, *DOR* duration of response, *EBC* early breast cancer, *ER* estrogen receptor, *ET* endocrine therapy, *HER2* human epidermal growth factor receptor 2, *HER2-E* HER2-enriched, *HR* hormone receptor, *iDFS* invasive disease-free survival, *IHC* immunohistochemical, *mAb* monoclonal antibody, *ORR* objective response rate, *OS* overall survival, *pCR* pathologic complete response, *PFS* progression-free survival, *pN0* pathologically node negative, *PR* partial response, *RCT* randomized controlled trial, *SERD* selective estrogen receptor degrader, *SD* stable disease, *SERM* selective estrogen receptor modulator, *TAM* tamoxifen, *TCHP* docetaxel, carboplatin, trastuzumab, and pertuzumab, *T-DM1* trastuzumab emtansin, *TKI* tyrosine kinase inhibitor, *TTP* time to progression.

### Early-stage breast cancer

#### Neoadjuvant studies testing combined receptor blockade targeting both HER2 and ER

Demonstration of meaningful clinical benefit from a combined receptor blockade strategy targeting HER2 and the ER has been challenging in the neoadjuvant setting, perhaps limited by the constraint of the limited number of therapeutic treatment cycles given in a preoperative setting in neoadjuvant study designs. Moreover, such trials frequently employ pCR as a primary clinical endpoint, whereas in adjuvant trial designs (with much longer duration of combined receptor blockade in the post-operative setting), time-to-event primary clinical endpoints (e.g., invasive disease-free survival [iDFS]) can be utilized.

In the randomized, phase III NSABP B-52 trial (NCT02003209), 315 patients with locally advanced, HR+/HER2+ invasive breast cancer received a 6-cycle regimen of neoadjuvant docetaxel, carboplatin, trastuzumab, and pertuzumab (TCHP) with or without estrogen deprivation (goserelin plus an AI) before undergoing surgery^76^. The primary endpoint of NSABP B-52 (pCR) was not met; no statistically significant difference in pCR (breast and lymph nodes) was observed between TCHP plus estrogen deprivation versus TCHP alone (46.1% vs. 40.9%; *P* = 0.36)^76^. However, with this trial design, the addition of chemotherapy to the dual HER2 antibody regimen may complicate assessment of the combined receptor blockade strategy of targeting HER2 and ER. For example, any advantage of combined receptor blockade against ER and HER2 could theoretically be offset by antagonism between chemotherapy and anti-estrogen treatment^98–101^. Moreover, the number of neoadjuvant cycles was limited to six in this trial. Longer exposure to combined receptor blockade strategies may be needed to observe beneficial clinical efficacy outcomes from this strategy. Importantly, no new safety signals emerged in NSABP B-52, with AEs reflecting expected additive toxicities from component agents of TCHP and ET. Grade 3/4 AEs included diarrhea (23%/<1% vs. 21%/0%), vomiting (8%/<1% vs. 5%/0%), and febrile neutropenia (5%/<1% vs. 7%/1%) for TCHP versus TCHP plus estrogen deprivation, respectively^76^.

In another neoadjuvant randomized clinical trial, the West German Study Group Adjuvant Dynamic Marker-Adjusted Personalized Therapy Trial Optimizing Risk Assessment and Therapy Response Prediction in Early Breast Cancer (WSG-ADAPT-HER2+) trial (NCT01817452), compared pCR rates of the HER2-directed antibody-drug conjugate ado-trastuzumab emtansine (T-DM1) versus trastuzumab with ET in early HER2+/HR+ breast cancer^91^. In this trial, 375 patients with early HER2+/HR+ breast cancer were randomized to receive a 12-week treatment of T-DM1 with or without ET or to trastuzumab with ET. The protocol-defined primary endpoint was pCR (defined as ypT0/is/ypN0). Previously, Rimawi et al. ^71^ had administered 12 weeks of trastuzumab, lapatinib, and ET with letrozole with or without a luteinizing hormone–releasing hormone agonist (dependent on menopausal status) to 64 patients. pCR (ypT0/is/ypN0) was 22% overall, 18% in ER+ disease, and 28% in ER− disease. Notably, the near-pCR percentage differed substantially between HR+ and HR− subtypes (54% vs. 40% with ypT1a/b or ypN0/is, respectively). In a subsequent trial with more prolonged (up to 24 weeks) preoperative therapy, the same researchers observed 33% pCR (breast only, ypT0/is) in ER+ disease versus 18% in ER−, following neoadjuvant treatment with lapatinib plus trastuzumab, with ET^94^. Building upon these results, this time with an antibody-drug conjugate (ADC) backbone (T-DM1) in the WSG-ADAPT HER+ trial, a pCR was observed in 41.0% of T-DM1–treated patients and 41.5% of T-DM1 plus ET-treated patients versus 15.1% of patients treated with trastuzumab and ET (*P* < 0.001)^91^. As in the case of the NSABP B52 trial, the authors acknowledge that one handicap of the WSG-ADAPT-HER2+ trial design is the use of just four preoperative cycles of T-DM1 ± ET, which may be insufficient to demonstrate a significant improvement in pCR by the addition of ET. In addition, we note that the derivative of maytansine (DM1) payload of T-DM1 is a microtubule-interacting chemotherapeutic payload, which in theory could also have antagonistic interactions with ET, thus negating potential synergistic interactions between ET and the trastuzumab antibody ADC backbone. In terms of mechanism of action, it is hypothesized that ADCs with cytotoxic payloads will follow all of the same principles as cytotoxic chemotherapeutics, perhaps including antagonism with anti-estrogens^102^. In terms of safety in WSG-ADAPT-HER2+, T-DM1 was associated with a significantly higher prevalence of grade 1 and 2 AEs, especially thrombocytopenia, nausea, and elevation of liver enzymes. Overall toxicity was low; seventeen therapy-related serious AEs (T-DM1 arms vs. trastuzumab plus ET; 5.3% vs. 3.1%, respectively) were reported^91^.

In another clinical trial that evaluated *duration* of neoadjuvant therapy in the context of HER2/HR combined receptor blockade, Masuda and colleagues^93^ designed a randomized, phase II, five-arm trial (UMIN-CTR identifier: UMIN000007576) to evaluate the efficacy and safety of lapatinib and trastuzumab (6 weeks) followed by lapatinib and trastuzumab plus weekly paclitaxel (12 weeks) with or without prolongation of anti-HER2 therapy prior to chemotherapy (18 vs. 6 weeks), and with or without ET in patients with HER2+ and/or ER+ disease. The primary endpoint was comprehensive pCR (CpCR) rate (CpCR included residual ductal carcinoma in situ of the breast). Although pCR with pN0 was achieved in 42.2% of 212 patients (ER−, 57.6%; ER+, 30.3%), there were no statistical differences based on *duration* of lapatinib and trastuzumab plus ET treatment (6 vs. 18 weeks)^93^. There were no major safety concerns associated with prolonging the anti-HER2 treatment or adding ET. Therefore, further research is required to support the benefit of longer-duration combination therapy with HER2-targeted agents and estrogen deprivation in maximizing clinical efficacy in the neoadjuvant setting. To this end, Prat Aparicio and colleagues have tested the hypothesis that biomarker selection of HER2+/HR+ patients may identify a subgroup with superior outcomes following combined receptor blockade (NCT01973660). Indeed, Prat Aparicio et al. ^92^ reported that after 18 weeks of trastuzumab plus lapatinib and ET, the overall pCR rate was just 18%, but with biomarker selection using the 50-gene PAM50 subtype classifier, the pCR rate was 31.6% in the HER2-enriched intrinsic subtype versus 5.3% for non-HER2 subtypes within the subset of HER2+/HR+ disease^92^. Indeed, further work by Pernas and Prat^103^ has shown that increased pCR rates in the PAM50 HER2-enriched gene expression phenotype are independent of HR expression^103^. Taken together, these observations hold promise that biomarker selection may be a path forward for patient identification for de-escalation treatment strategies utilizing combined receptor blockade, potentially including non-chemotherapy regimens, for carefully selected patients with early-stage HER2+. However, at the time of this writing, neither the National Comprehensive Cancer Center Network (NCCN) guidelines nor the American Society of Clinical Oncology (ASCO) guidelines have as yet recognized the PAM50 assay as a biomarker for patient selection for neoadjuvant therapy considerations in early-stage HER2+ disease. Interestingly, we note that Prat and colleagues^104^ have recently reported development and validation of a new assay (HER2DX) for predicting pathological response and survival outcome in early-stage HER2+ breast cancer^104^. HER2DX is a supervised learning algorithm that generates a single score based on tumor size, nodal status, and four-gene expression signature that tracks immune infiltration, cell proliferation, luminal differentiation, and HER2 expression. Using both training and validation clinical cohorts, the investigators have shown that HER2DX variables were significantly (*P* = 0.002) associated with good risk outcomes (i.e., immune and luminal) and poor risk outcomes (i.e., proliferation and tumor and nodal stage), with the 5-year DFS in the low-risk group being 97.4%. For a neoadjuvant training cohort, HER2DX variables were associated with pCR (i.e., immune, proliferation, and HER2 amplicon) and non-pCR (i.e., luminal and tumor and nodal staging), with continuous HER2DX pCR likelihood score significantly associated with pCR (*P* < 0.0001). However, we note that HER2DX has not yet been tested or validated as a predictor of clinical efficacy from a combined receptor (HER2/ER) blockade treatment strategy^104^.

Finally, as noted in the preclinical section above, convergence of HER2 and ER signals on RB1 suggests that a combined pharmacological intervention with individual drugs directed to all three targets (HER2, ER, and RB1) could be synergistic. In the open-label NA-PHER2 trial (NCT02530424), the CDK4/6 inhibitor palbociclib was added to trastuzumab, pertuzumab, and fulvestrant in the neoadjuvant setting and resulted in an objective clinical response in 29 of 30 patients (97%, before surgery)^69^. The most frequent grade 3 AEs in NA-PHER2 were neutropenia (29%), diarrhea (14%), and stomatitis; increased alanine aminotransferase levels; and hypersensitivity reactions (3% of each event). No grade 4 or serious AEs were recorded in the trial, and there were no deaths. Additionally, the combination had a significant effect on the expression of Ki67 at 2 weeks following treatment initiation; and at surgery, eight (27%; 95% CI, 12–46%) patients had a pCR in breast and axillary nodes^69^. Thus, in addition to biomarker selection strategies such as PAM50, more complete coverage of HR-HER2 crosstalk and associated downstream signaling (in this case, by the addition of CDK4/6 inhibition) may offer future clinical consideration of chemotherapy-free regimens as neoadjuvant treatment for HR+/HER2+ early breast cancer. In summary, the need for these newer approaches is underscored by results from the neoadjuvant WSG-TP-II trial results. In the prospective WSG TP-II phase II trial (NCT03272477), 207 patients with centrally confirmed HR+/HER2+ early breast cancer were randomized to 12 weeks of standard ET (*n* = 100) versus paclitaxel 80 mg/m^2^ weekly (*n* = 107) plus trastuzumab and pertuzumab intravenously every 3 weeks; all patients received dual HER2-antibody blockade in the adjuvant setting. The primary endpoint was pCR (ypT0/is/ypN0), which was observed in 24% (95% CI, 16–34%) in patients with ET plus the dual anti-HER2 antibodies versus 57% (95% CI, 47–67%) in patients receiving paclitaxel chemotherapy plus trastuzumab and pertuzumab (odds ratio, 0.24; 95% CI, 0–0.46; *P* < 0.001). Neoadjuvant treatment was well tolerated in both trial arms and completed per protocol in 93 and 92 patients in the ET plus pertuzumab plus trastuzumab and the paclitaxel plus pertuzumab plus trastuzumab) arms, respectively. Only nine and thirteen serious AEs, respectively, were reported in each group during neoadjuvant therapy^90^. Thus with the chemotherapy-based neoadjuvant regimen yielding significantly superior pCR, and the expectation that pCR in HER2+ early breast cancer will correlate with long-term time-to-event clinical outcomes (i.e., iDFS and overall survival [OS])^105–107^, the current standard of care for HER2+/HR+ early breast cancer at this time (for the vast majority of patients and outside the context of participation in a clinical trial) remains a combination of chemotherapy plus dual anti-HER2 antibodies.

#### Combined receptor blockade targeting HER2 and ER in the adjuvant and extended adjuvant setting

The most common setting in which HER2-blockade and ET are combined is in the adjuvant setting. Because the optimal adjuvant ET for HER2+/HR+ patients remains unclear, investigators in the Short-HER trial (853 patients with HR+ breast cancer were included) evaluated the impact of ET type on DFS in patients with HER2+/HR+ breast cancer (NCT00629278). After a median follow-up of 8.7 years, patients who received AIs had a significantly better DFS versus patients on tamoxifen or tamoxifen followed by an AI (7-year DFS, 87.3% vs. 81.7%; log-rank *P* = 0.017; hazard ratio, 1.46, 95% CI, 1.05–2.03). In multi-variate analyses including menopausal status, stage, and treatment arm, the type of ET maintained a significant association with DFS. Additionally, the use of gonadotropin-releasing hormone was associated with numerically improved DFS in premenopausal patients (86.6% vs. 81.6%; log-rank *P* = 0.168; hazard ratio, 0.70; 95% CI, 0.43–1.16). Although there are no randomized trials of ET versus not in the adjuvant setting for HER2+ early-stage breast cancer in the post-trastuzumab era, the observation that the specific type of ET in the Short-HER trial is significantly associated with long-term time-to-event clinical outcomes (i.e., DFS) *strongly* suggests that ET is impactful (compared to omission of anti-estrogen treatment) in patients with HER2+ early-stage disease in the adjuvant setting^88^. Indeed, the combined receptor blockade strategy targeting HER2 and ER is now being investigated in a non-chemotherapy (de-escalation trial design) adjuvant treatment in an ongoing phase II trial of adjuvant ET, pertuzumab, and trastuzumab (administered subcutaneously with recombinant hyaluronidase) in patients (*N* = 375) with anatomic stage I HR+/HER2+ breast cancer (ADEPT; NCT04569747).

One strategy to overcome resistance to HER2-targeted therapy is to extend the duration of adjuvant therapy, with the addition of an irreversible pan-HER TKI, neratinib, taken for 1 year. In the randomized, placebo-controlled, phase III ExteNET trial (NCT00878709), the effect of neratinib (240 mg/day) was evaluated in patients (*n* = 2840) with stage I-III HER2+ breast cancer who had completed neoadjuvant and adjuvant chemotherapy plus trastuzumab up to 2 years prior to randomization; concurrent adjuvant ET was recommended for patients with HR+ disease^7^. The inclusion criteria were subsequently amended to recruit higher-risk patients (defined as patients with stage II-III node-positive disease who had completed trastuzumab therapy up to 1 year prior to randomization)^7^. At the 2-year follow-up, iDFS rate was 93.9% in patients receiving neratinib versus 91.6% in those receiving placebo (stratified hazard ratio, 0.67; 95% CI, 0.50–0.91; *P* = 0.0091)^7^. This benefit was maintained at the 5-year analysis of ExteNET, with 5-year iDFS rates favoring neratinib (90.2% vs. 87.7%; stratified hazard ratio, 0.73; 95% CI, 0.57–0.92; *P* = 0.0083) after a median follow-up of 5.2 years^79^. A prespecified subgroup analysis suggested that the iDFS benefit of neratinib was more pronounced in patients with HR+ tumors at 2 years (hazard ratio, 0.51; 95% CI, 0.33–0.77; *P* = 0.0013) and at 5 years (hazard ratio, 0.60; 95% CI, 0.43–0.83)^7,79^ versus HR− tumors at the 2-year (hazard ratio, 0.93; 95% CI, 0.60–1.43; *P* = 0.74) and 5-year time points (hazard ratio, 0.95; 95% CI, 0.66–1.35)^7,79^. After a median follow-up of 8 years, reduced OS hazard ratios (0.95–0.78) upon completion of neratinib therapy further supported the benefit of neratinib treatment. Such benefit was also observed across subgroups with HR+ tumors^108^. It is postulated that the enhanced efficacy of neratinib in HR+/HER2+ breast cancer in the ExteNET trial *may* be explained by interruption of crosstalk between the ER and HER2 pathways given that >90% of patients with HR+ disease in ExteNET received concurrent adjuvant ET along with extended adjuvant neratinib. In support of this hypothesis, the ExteNET investigators have published a subset analysis on “patients of clinical interest” in ExteNET who: (1) were HR+, (2) were enrolled within 1 year of completion of adjuvant trastuzumab treatment, 3) had received prior neoadjuvant therapy, and 4) had not achieved a pCR. In this small subset (*N* = 295), 8-year OS rates were 91.3% (95% CI, 84.4–95.2%) in the neratinib group versus 82.2% (95% CI, 75.1–87.4%) for placebo, corresponding to a 9.1% absolute benefit in OS (hazard ratio, 0.47; 95% CI, 0.23–0.92)^89^. Rightly, no *P-*values were published for this retrospective, highly selected exploratory subset with modest sample size. Moreover, due to constraints in the trial design (in particular, no neratinib-alone control arm without ET), it is not scientifically possible to draw definitive conclusions regarding mechanistic insight for clinical outcome observations in the HR+ subgroup(s) of ExteNET. It is important to note that due to ethical considerations of withholding ET, inclusion of such control groups is not clinically feasible in many of the clinical trials conducted to date testing the combined receptor blockade hypothesis. Thus, it is not possible to state definitively that the ExteNET trial results *prove* the combined receptor blockade (HER2/ER) hypothesis. Instead, it is only possible to state that the trial results are consistent with the hypothesis. In the United States, neratinib is approved in the extended adjuvant setting in HER2+ early breast cancer irrespective of HR status, based upon the intent-to-treat principle; however, the European Medicines Agency has approved neratinib only for HR+/HER2+ patients^109^. In the ExteNET trial, diarrhea was observed in 95% of patients, with grade 3 diarrhea observed in 40% of patients. In addition, a considerable proportion of patients experienced nausea, abdominal pain, fatigue, and vomiting (NERLYNX^®^ (neratinib) tablets, for oral use [package insert]. Los Angeles, CA: Puma Biotechnology, Inc.; 2022). It is important to note that neratinib-associated diarrhea may be mitigated simply by gradually increasing the dose over time (120 mg/day Days 1–7, 160 mg/day Days 8–14, and 240 mg/day thereafter and loperamide administered as needed), as demonstrated in the CONTROL clinical trial (NCT02400476)^110^.

### Combined receptor blockade targeting HER2 and ER in advanced (locally recurrent, surgically unresectable or metastatic) breast cancer

Because HER2-positivity in HR+ breast cancer is associated with ET resistance, potentially due to the crosstalk mechanism(s), including attenuation of ER and PR expression^8^, hormone sensitivity may theoretically be restored through HER2 blockade^111^. Early randomized phase III trials demonstrated a clinical benefit for combining an AI (anastrozole or letrozole) with trastuzumab or lapatinib in patients with ER+/HER2+ metastatic breast cancer^65–67^. In the randomized phase III TAnDEM trial (NCT00022672), the safety and efficacy of anastrozole plus trastuzumab was evaluated in patients with HR+/HER2+ metastatic breast cancer. Results showed that patients in the trastuzumab plus anastrozole arm experienced significant progression-free survival (PFS) improvements compared with patients receiving anastrozole alone (hazard ratio, 0.63; 95% CI, 0.47–0.84; median PFS, 4.8 vs. 2.4 months; log-rank *P* = 0.0016)^67^. In TAnDEM, grade 3 and 4 AEs occurred in 23% and 5% of patients, respectively, in the trastuzumab plus anastrozole arm, and 15% and 1% of patients, respectively, in the anastrozole-alone arm; there was one patient who experienced New York Heart Association class II congestive heart failure in the combination arm^67^. However, interpretation of these results is limited by the lack of a trastuzumab-alone control arm. Nevertheless, the benefit from the addition of a HER2-inhibiting agent to an AI was confirmed with the open-label, randomized, phase III eLEcTRA trial (NCT00171847), in which numerically greater improvements in time to progression (14.1 vs. 3.3 months), objective response rate (ORR; 27% vs. 13%), and clinical benefit rate (CBR; 65% vs. 39%) were observed with letrozole plus trastuzumab versus letrozole alone^73^. In eLEcTRA, most AEs were classified as Common Terminology Criteria grade 1 or 2. Cardiac events were comparable in all arms (8.6–9.7%), with no life-threatening (grade 4) events reported^73^.

The combination of letrozole plus lapatinib was evaluated in the phase III EGF 30008 trial (NCT00073528) of patients with HR+ metastatic breast cancer. In 219 HER2+ patients, combination therapy improved median PFS (the trial primary endpoint) to 8.2 months compared with 3 months for letrozole alone (hazard ratio, 0.71; 95% CI, 0.53–0.96; *P* = 0.019)^65^. Overall response rate and CBR rate were also significantly improved with letrozole plus lapatinib (Table 2), garnering US Food and Drug Administration approval for the combination in 2010. Grade 3 or 4 AEs were more common in the lapatinib-letrozole arm versus letrozole-placebo arm (diarrhea, 10% vs. 1%; rash, 1% vs. 0%, respectively), but they were considered manageable^65^. The trial also included a large cohort (*N* = 952) of HR+/HER2− patients to determine whether the EGFR kinase inhibitory effects of lapatinib (along with HER2 kinase inhibition in a HER2− population) would also synergize with aromatase inhibition. This effect was *not* observed, with no improvement in PFS and no statistically significant difference in response rates^65^. Thus, combined receptor blockade targeting EGFR and HER2 and ER in HER2−/HR+ patients was unsuccessful in a large prospective randomized phase III clinical trial.

Dual HER2 inhibition with synergistic combination of lapatinib and trastuzumab was studied in combination with an AI, without chemotherapy, in the phase III ALTERNATIVE trial (NCT01160211) of patients with HR+/HER2+ metastatic breast cancer who had been treated with prior trastuzumab and ET^112^. Median PFS was significantly improved by 38% with lapatinib, trastuzumab, and AI (11 months) versus trastuzumab and AI (5.6 months; hazard ratio, 0.62; 95% CI, 0.45–0.88; *P* = 0.0063)^95^. This effect on PFS was observed across predefined subgroups of patients with measurable disease, patients treated with AIs, and patients who had previously received trastuzumab. Overall response rate and CBR also favored the three-drug combination regimen^95^. In ALTERNATIVE, common AEs (≥15%) with lapatinib plus trastuzumab plus AI, trastuzumab plus AI, and lapatinib plus AI were diarrhea (69%, 9%, and 51%, respectively), rash (36%, 2%, and 28%, respectively), nausea (22%, 9%, and 22%, respectively), and paronychia (30%, 0%, and 15%, respectively), most of which were grade 1 or 2. The incidence of serious AEs was similar across groups, and AEs leading to discontinuation were actually lower with lapatinib plus trastuzumab plus AI^95^.

The combination of neratinib plus ET has also been assessed in patients with *ERBB2*-*mutated*, non-amplified metastatic breast cancers^63,96,113–115^. Such mutations most frequently involve the kinase domain and are constitutively activating. The SUMMIT trial (NCT01953926) is an open-label phase II multi-histology “basket” trial evaluating the efficacy of neratinib in patients with a variety of tumors harboring *ERBB2* or *ERBB3* gene mutations, including breast, lung, bladder, biliary, cervical, and colorectal cancers^113,114^. The breast cancer cohort of SUMMIT comprised heavily pretreated patients (*n* = 81) with metastatic *ERBB2*-mutant breast cancer who were treated with neratinib monotherapy (240 mg/day; *n* = 34, including 23 with ER+ breast cancer) or neratinib (240 mg/day) plus fulvestrant (500 mg on Days 1 and 15 of Month 1, then on Day 1 every 4 weeks; *n* = 47)^79–96^. Promising antitumor activity was observed, with a confirmed ORR (primary endpoint) of 17% (95% CI, 5–39%) for patients with ER+ tumors in the neratinib monotherapy group and 30% (95% CI, 17–45%) for neratinib plus fulvestrant; CBRs were 30% (95% CI, 13–53%) and 47% (95% CI, 32–62%), respectively^96,113,114^. As the SUMMIT trial data met the original objective of evaluating the use of neratinib in *ERBB2*-mutant tumors, trial enrollment has recently concluded. The five most common AEs (all grades) reported in SUMMIT were diarrhea (90.2%, necessitating high-dose loperamide prophylaxis), nausea (72.5%), vomiting (52.9%), fatigue (43.1%), and constipation (41.2%)^87^. In another trial (part 1 of the open-label MutHER trial (NCT01670877), 16 patients with heavily pretreated metastatic breast cancer (15 of whom had ER+/HER2+ tumors) received neratinib monotherapy^63^. A total of five patients (31%) experienced clinical benefit. In part 2 of the trial, 31 patients with HR+/*ERBB2*-mutated metastatic breast cancer received neratinib plus fulvestrant, with a CBR of 40% (95% CI, 19–64%) in patients previously treated with fulvestrant and 30% (95% CI, 7–65%) in fulvestrant-naive patients^97^. Median PFS was 24 weeks (95% CI, 16-31) and 20 weeks (95% CI, 8-not assessed) in fulvestrant-exposed and fulvestrant-naive patients, respectively. The phase II MutHER trial has recently been updated^83^. The investigators evaluated the efficacy of neratinib plus fulvestrant in patients with ER+/*ERBB2*-mutant, *ERBB2* non-amplified metastatic breast cancer in a fulvestrant-treated (*n* = 24) or fulvestrant-naive cohort (*n* = 11). Patients with ER−/*ERBB2* mutant metastatic breast cancer received neratinib monotherapy in a small exploratory cohort (*n* = 5). The CBR was 38% (95% CI, 18–62%), 30% (95% CI, 7–65%), and 25% (95% CI, 1–81%) in the fulvestrant-treated, fulvestrant-naive, and ER− cohorts, respectively. Anecdotally, adding trastuzumab at progression in five patients resulted in three objective partial clinical responses and one stable disease of ≥24 weeks duration. CBR appeared positively associated with lobular histology and negatively associated with *ERBB2* L755 alterations. Additional acquired *ERBB2* mutations were detected in five of 23 patients at progression^83^. Taken together, these data suggest that the concept of combined receptor blockade targeting HER2 and ER extends beyond clinical HER2+ tumors (defined as HER2 gene amplification and/or 3+ immunohistochemical [IHC] protein overexpression) to include *ERBB2*-mutant biology, the latter very likely mimicking constitutive activation of the HER2 kinase, as is seen in cases of aberrant HER2 overexpression. The safety profile of neratinib or neratinib plus fulvestrant was consistent with prior trials. Across all cohorts, the most common AEs considered related to the trial were diarrhea (85%, with high-dose loperamide prophylaxis), nausea (53%), fatigue (50%), anorexia (35%), and aspartate aminotransferase level increase (28%). Neratinib was dose-reduced in six (15%) patients who received neratinib 200 mg daily plus fulvestrant due to nausea/vomiting (*n* = 2), diarrhea (*n* = 3), or elevated liver enzyme levels (*n* = 1). One patient had a second dose reduction to 160 mg daily due to diarrhea. No patients discontinued therapy due to AEs^83^.

In terms of other agents, previous findings have shown that dual CDK4 and CDK6 inhibitors could sensitize HER2-targeted therapies and delay tumor recurrence in HER2+ breast cancer models^81^. In a phase Ib/II trial (NCT03054363), the combination of tucatinib plus palbociclib plus letrozole is under investigation for the treatment of patients with HR+/HER2+ metastatic breast cancer^80^. The interim analysis results, after a median follow-up of 6 months in 40 patients, showed that the combination was well tolerated with manageable and expected AEs. The most common grade ≥3 AEs were neutropenia (60%), leukopenia (24%), diarrhea (19%), fatigue (14%), and infections (14%). The median PFS was 8.7 months, with 10.1 months for patients without brain metastasis and 6.0 months for those with brain metastasis^116^. Another CDK4/6 inhibitor, SHR6390, is being investigated in a phase Ib/II trial (NCT03772353) in combination with pyrotinib and letrozole in patients with HR+/HER2+ metastatic breast cancer. In the 15 patients enrolled to date, 10 patients had achieved a confirmed partial response and four patients had stable disease. Enrollment is ongoing and further results, including safety signals, will be reported^81^. In a randomized, open-label, phase II trial (MonarcHER; NCT02675231), the CDK4/6 inhibitor abemaciclib was assessed in patients with HR+/HER2+ advanced breast cancer (recurrent locally advanced, unresectable, or metastatic disease). Patients were randomized 1:1:1 to one of three treatment arms: oral abemaciclib 150 mg BID, IV trastuzumab (8 mg/kg on cycle 1 followed by 6 mg/kg thereafter) and IM fulvestrant 500 mg (arm A); abemaciclib plus trastuzumab (arm B); or standard of-care single agent physician’s choice chemotherapy plus trastuzumab (arm C). Results showed that after a median follow-up of 52.9 months,157 mortality events had occurred across the arms: 63%, 68% and 67% of patients in arms A, B, and C, respectively. Median OS was 31.1 months in arm A, 29.2 months in arm B, and 20.7 months in arm C (arm A vs. arm C: hazard ratio, 0.75; 95% confidence interval [CI] 0.47–1.21; *P* = 0.243; arm B vs. arm C: hazard ratio, 0.73; 95% CI 0.46–1.15; *P* = 0.177)^117^. Earlier published results showed a significant PFS benefit in patients who received abemaciclib, trastuzumab, and fulvestrant (8.3 months; 95% CI, 5.9–12.6) compared with patients who received standard-of-care chemotherapy and trastuzumab (5.7 months; 5.4–7.0; hazard ratio, 0.67 [95% CI, 0.45–1.00]; *P* = 0.051)^37^. Abemaciclib was generally well tolerated in monarcHER; however, the incidence of thrombocytopenia was higher than that previously reported, perhaps because >96% of the patients in monarcHER had been treated with prior T-DM1^37^. This concept will be tested further in early-stage breast cancer in the ongoing eMonarcHER trial: a randomized, double blind, placebo-controlled phase III trial (*N* = 2450) of abemaciclib plus standard adjuvant endocrine therapy in participants with high-risk, node-positive, HR+/HER2+ early breast cancer who have completed adjuvant HER2-targeted therapy (NCT047523320). In the SOLTI-1303 PATRICIA trial (NCT02448420; *N* = 72), palbociclib was assessed in combination with trastuzumab with or without ET in patients with HER2+ advanced breast cancer. The median number of prior lines of systemic therapy for metastatic disease was three. Interestingly, among HR+/HER2+ patients in PATRICIA, PAM50 intrinsic subtype was significantly associated with PFS in patients with ER+ disease (*P* = 0.001 by log-rank test). Median PFS was 10.6 months (95% CI, 4.1–14.8) in Luminal B, 8.2 months (95% CI, 2.2–24.1) in Luminal A, 3.8 months (95% CI, 2.1–10.9) in HER2-E, and 6.0 months (95% CI, 1.7–11.2) in normal-like. In univariate analysis, patients with luminal disease had better PFS than patients with non-luminal tumors (median PFS 10.6 vs. 4.2 months; hazard ratio, 0.32; 95% CI, 0.25–0.98; *P* = 0.0020). In PATRICIA, 64.8% of patients reported a grade 3 or higher hematologic treatment-related AE, with 63.4% and 11.3% reporting grade 3 or higher neutropenia and thrombocytopenia, respectively. Three patients (4.2%) reported febrile neutropenia. Grade 3 or higher non-hematologic treatment-related AEs occurred in 7% of patients, most commonly grade 3 asthenia in two patients. No clinically significant cardiovascular toxicity was observed^86^. Finally, the PATINA phase III trial (NCT02947685) will evaluate the combination of palbociclib, anti-HER2 therapy, and ET in patients with HR+/HER2+ metastatic breast cancer as a maintenance strategy following four to eight cycles of induction treatment with taxane chemotherapy plus anti-HER2 antibodies pertuzumab plus trastuzumab^118^. It is hoped that the results of these trials may translate the preclinical hypothesis that integration of CDK4/6 inhibition along with anti-HER2 agents and anti-ER will result in a therapeutic advantage in the clinical domain and, in some cases, offer non-chemotherapeutic regimens as effective alternative treatment options.

**Fig. 2:**
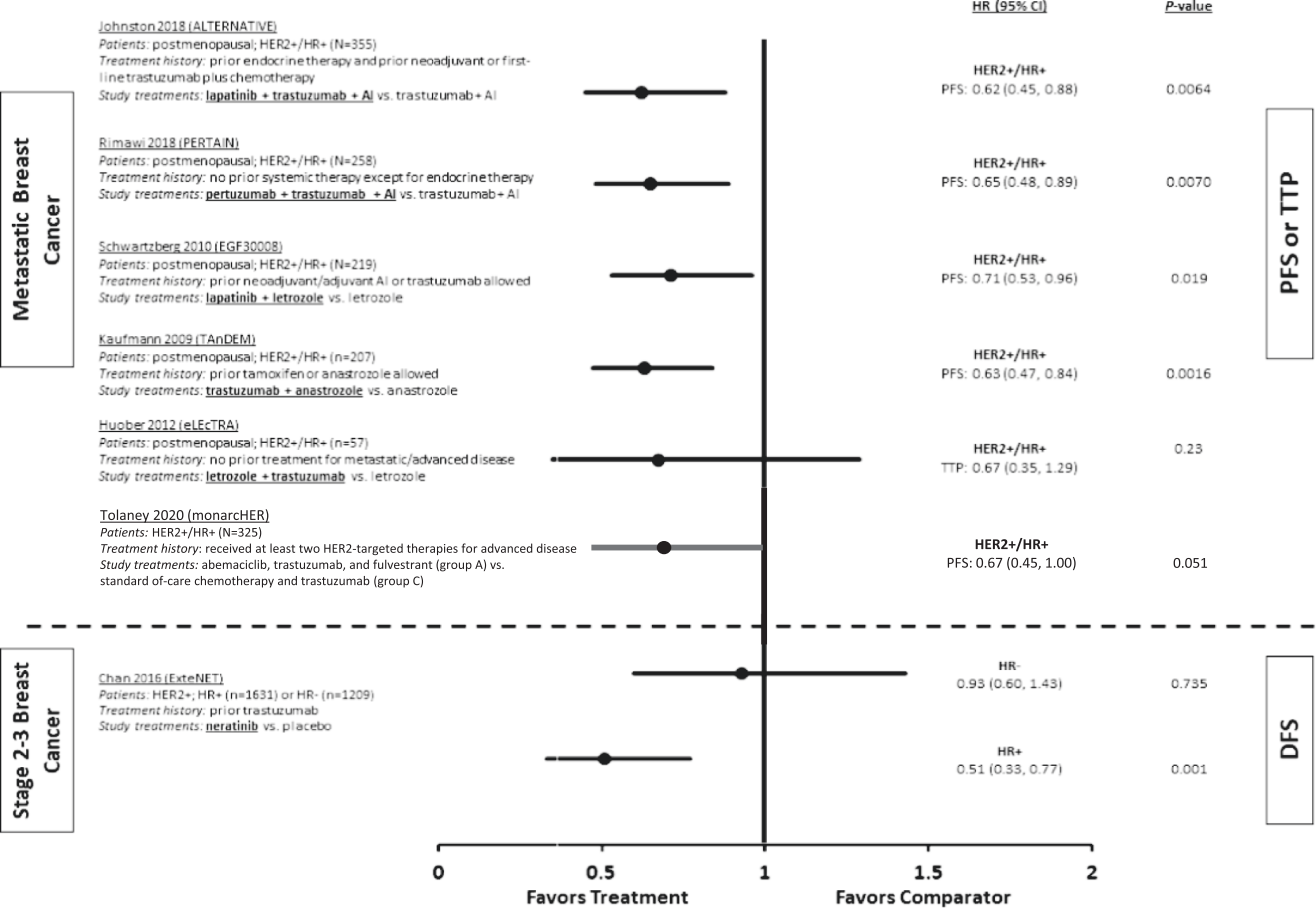
PFS and DFS benefits of dual ER/HER2 blockade in key phase 2 and 3 clinical trials. Error bars indicate 95% CI. AI aromatase inhibitor; CI confidence interval; DFS disease-free survival; ER estrogen receptor; HER2 human epidermal growth factor receptor 2; HR hormone receptor; PFS progression-free survival; TTP time to progression.

Findings from these clinical trials indicate that dual blockade of ER and HER2 is an effective treatment approach across the continuum of HR+/HER2+ breast cancer disease settings (Fig. 2)^7,38,66–68,73^. These combination treatment strategies can improve clinical outcomes in HR+/HER2+ or *ERBB2*-mutant breast cancer, potentially overcoming resistance to ET arising from crosstalk between the ER and HER2 signaling pathways.

Results from other trials suggest that bi-directional signaling pathways are also evident between the ER and downstream PI3K/AKT/mTOR signaling pathways, which may play an important role in ER+ breast cancers^119,120^. The PI3K/AKT/mTOR signaling pathway drives many cellular processes, including proliferation, growth, and survival. Aberrant signaling of this pathway has been implicated in the tumorigenesis of ER+ breast cancer^121–123^ and development of endocrine resistance^124–126^. Inhibition of the PI3K/AKT/mTOR signaling pathway has been shown to augment the response of ER+ breast cancer to ET, indicating that dual targeting of the ER and PI3K/AKT/mTOR pathways may be another rational treatment approach^119,120^. In contrast, combinations of anti-HER2 therapy and mTOR inhibition have yielded only minimal clinical activity in the metastatic setting, at the expense of enhanced toxicity (as reviewed by Holloway and Marignani^127^ and Fujimoto et al. ^128^). A two-part phase III trial (a safety run-in cohort, followed by a randomized placebo-controlled cohort) is ongoing (EPIK-B2; NCT04208178) to evaluate the efficacy and safety of alpelisib, a PI3K inhibitor, in combination with dual HER2 inhibition with trastuzumab and pertuzumab in patients with HER2+ (any HR status) advanced breast cancer in the “maintenance” setting (a similar trial design as in the PATINA clinical trial, *cf*. Above) following completion of taxane chemotherapy combined with dual HER2 antibody therapy^129^.

Finally, to underscore the potential clinical utility of combined receptor blockade, in the SYSUCC-002 clinical trial, Hua and colleagues^85^ tested whether trastuzumab plus ET is *non-inferior* to trastuzumab plus chemotherapy in an open-label, non-inferiority, phase III, randomized, controlled trial (NCT01950182) in the first-line metastatic disease setting, at nine hospitals in China. Patients in SYSUCC-002 were randomly assigned (1:1) to receive trastuzumab plus ET (per investigator’s choice of ER modulators or AI, with or without concurrent ovarian suppression) or chemotherapy (per investigator’s choice of taxanes, capecitabine, or vinorelbine). The primary endpoint was PFS with a hazard ratio non-inferiority upper margin of 1.35. In 392 patients, after a median follow-up of 30.2 months, the median PFS was 19.2 months (95% CI, 16.7–21.7) in the ET group and 14.8 months (12.8–16.8) in the chemotherapy group (hazard ratio, 0.88; 95% CI, 0.71–1.09; non-inferiority *P* <0.0001). Not surprisingly, toxicity was observed significantly more frequently in the chemotherapy group compared with the ET group. Most AEs in the ET group were grade 1 to 2. The most common AEs were joint pain (16.8%), muscle pain (16.3%), and fatigue (15.8%). The most frequently reported AEs in the chemotherapy group were alopecia (63.8%), leukopenia (50.0%), and nausea (47.5%). Patients in the ET group had a significantly lower prevalence of AEs of grade 3 to 4 compared with those in the chemotherapy group (3.1% vs. 51.0%; *P* < 0.01). This provocative result suggests that, as in the case of HER2−/HR+ metastatic breast cancer, in which usually multiple endocrine manipulations are utilized prior to consideration of palliative chemotherapy regimens, the same principle may apply even in the context of HER2+/HR+ disease. However, the SYSUCC-002 trial has limitations in that the hazard ratio upper bound defining non-inferiority was rather modest (1.35) and the trial did not incorporate pertuzumab in the first-line HER2+ metastatic setting, which is the current standard of care (based upon a large and highly significant OS advantage in first-line HER2+ metastatic disease in the CLEOPATRA trial^84^) and only a minority of patients in SYSUCC-002 were assigned to taxane chemotherapy in the control arm. Thus, overall, such intriguing results from SYSUCC-002 notwithstanding, further clinical trials are warranted to optimize patient selection, identify novel biomarkers, and improve those currently available to guide therapy, optimize existing combinations, and investigate novel targeted therapies that have the potential to overcome endocrine and anti-HER2 treatment resistance in the metastatic setting^40^.

## Summary

Preclinical studies performed since 1995 have provided compelling evidence of a bi-directional molecular crosstalk between the ER and HER2 cellular signaling pathways that promote tumor growth and progression^36^. The use of combined anti-HER2 therapy and ET needs to be addressed because it is an underestimated (and underutilized) option in both settings of early breast cancer and metastatic breast cancer. Further support for crosstalk came from studies that showed treatment strategies targeting a single pathway (ER or HER2) resulted in the upregulation of the other pathway, ultimately resulting in resistance to therapy. These findings led to assessment of simultaneous ER and HER2 blockade in ER+/HER2+ breast cancer models, in which enhanced antitumor activity was observed, compared with single-target blockade controls^36,42^.

In the clinical setting, various combinations targeting the ER and HER2 signaling pathways have been evaluated, and the evidence to date suggests that this approach produces enhanced and sustained antitumor activity compared with targeting either pathway alone (with the aforementioned caveat that many trial designs lacked single-agent–alone control groups for *either* HER2- or ER-targeting agents). Clinical trials have shown an enhanced benefit with combined endocrine and HER2-directed therapies in patients with HR+/HER2+ or *ERBB2*-mutated breast cancers. Additional research is needed to identify patient subpopulations that will derive optimal clinical benefit from currently available treatment approaches, as well as new combination treatment strategies and novel agents with downstream effects that may improve clinical outcomes^69,92^. Moreover, a revolution in the field of HER2-targeting has recently happened, with the emergence of the targetability of “HER2-low” (tumors with HER2 immunohistochemical staining of 1+ or 2+ without evidence of *ERBB2* gene amplification by in situ hybridization) breast cancer with fam-trastuzumab deruxtecan (an antibody-drug conjugate composed of a humanized anti-HER2 monoclonal antibody and a topoisomerase I inhibitor payload). In the randomized phase III DESTINY-Breast04 trial (NCT03734029), patients with one or two prior lines of chemotherapy for advanced/metastatic disease were randomly assigned (2:1) to receive trastuzumab deruxtecan or the physician’s choice of chemotherapy. A total of 494 of 557 randomly assigned patients (88.7%) had HR+ disease. The median PFS (primary endpoint) in this cohort was 10.1 months in the trastuzumab deruxtecan group and 5.4 months in the physician’s choice group (hazard ratio for disease progression or death, 0.51; *P* < 0.001), and OS was 23.9 months and 17.5 months, respectively (hazard ratio for death, 0.64; *P* = 0.003)^130^. Interestingly, in contrast to the quantitatively inverse correlation between *ERBB2* gene amplification and ER/PR protein expression published by Konecny et al. ^8^, ER and HER2 expression is *positively* correlated in HER2− tumors^131^. In a recent report, HR expression was significantly more common among HER2-low compared with HER2-IHC zero breast cancer (89.9% vs. 80.9%; *P* < 0.001)^132^. These findings may be important to consider in future clinical trials of anti-HER2 and anti-ET in HER2− (including HER2-low) disease. For example, one such trial is the ongoing DESTINY-Breast08 trial (NCT04556773) of fam-trastuzumab deruxtecan plus ET with either anastrozole or fulvestrant^133^.

In conclusion, sustained inhibition of either HER2 or ER can result in functioning of the other pathway as a key means of escape/survival in ER+/HER2+ breast cancer cells. Combined receptor (HER2 plus ET) blockade may help overcome the resistance to therapy in this disease population^8,24–28^, potentially reduce the use of chemotherapy^69^, and hopefully improve survival results^89,117^ while maintaining clinical feasibility in terms of safety and tolerability. Further research in patient selection, predictive biomarkers^92^, treatment sequencing^7,118,129^, and combination therapies^118–129^, as well as novel targeted therapies, are needed in this population to improve patient outcomes.

### Reporting summary

Further information on research design is available in the Nature Research Reporting Summary linked to this article.

## Supplementary information

Reporting Summary
